# To Explore the Pathogenesis of Vascular Lesion of Type 2 Diabetes Mellitus Based on the PI3K/Akt Signaling Pathway

**DOI:** 10.1155/2019/4650906

**Published:** 2019-04-17

**Authors:** Jia-Rong Gao, Xiu-Juan Qin, Zhao-Hui Fang, Li-Shan, Li-Ping Han, Hui-Jiang, Ming-Fei Guo, Nan-Nan Jiang

**Affiliations:** ^1^Department of Pharmacy, The First Affiliated Hospital of Anhui University of Chinese Medicine, 117 Meishan Road, Hefei, China; ^2^Department of Nephrology, The First Affiliated Hospital of Anhui University of Chinese Medicine, 117 Meishan Road, Hefei, China

## Abstract

**Background:**

Type 2 diabetes mellitus (T2DM) has become a chronic disease, serious harm to human health. Complications of the blood pipe are the main cause of disability and death in diabetic patients, including vascular lesions that directly affects the prognosis of patients with diabetes and survival. This study was to determine the influence of high glucose and related mechanism of vascular lesion of type 2 diabetes mellitus pathogenesis.

**Methods:**

In vivo aorta abdominalis of GK rats was observed with blood pressure, heart rate, hematoxylin and eosin (H&E), Masson, and Verhoeff staining. In vitro cells were cultured with 30 mM glucose for 24 h. RT-QPCR was used to detect the mRNA expression of endothelial markers PTEN, PI3K, Akt, and VEGF. Immunofluorescence staining was used to detect the expression of PTEN, PI3K, Akt, and VEGF. PI3K and Akt phosphorylation levels were detected by Western blot analysis.

**Results:**

Heart rate, systolic blood pressure, diastolic blood pressure, and mean blood pressure in the GK control group were higher compared with the Wistar control group and no difference compared with the GK experimental model group. Fluorescence intensity of VEGF, Akt, and PI3K in the high-sugar stimulus group was stronger than the control group; PTEN in the high-sugar stimulus group was weakening than the control group. VEGF, Akt, and PI3K mRNA in the high-sugar stimulus group were higher than the control group; protein expressions of VEGF, Akt, and PI3K in the high-sugar stimulus group were higher than the control group. PTEN mRNA in the high-sugar stimulus group was lower than the control group. Protein expression of PTEN in the high-sugar stimulus group was lower than the control group.

**Conclusions:**

Angiogenesis is an important pathogenesis of T2DM vascular disease, and PTEN plays a negative regulatory role in the development of new blood vessels and can inhibit the PI3K/Akt signaling pathway.

## 1. Introduction

Diabetes is a common chronic disease with enhanced glucose levels occurring in the long term, which have an impact on the body and exert a number of negative effects [1]. In diabetes mellitus, glucose lipid metabolism disorders, ischemia hypoxia, inflammatory stimulation, and growth factors all promote angiogenesis. Atherosclerosis is a type of chronic pathological change in the middle arteries, which is the basis of the formation of diabetic vascular complications. Vascular endothelial dysfunction is considered to be the earliest manifestation of atherosclerosis and is a necessary initial stage of atherosclerosis [2, 3]. One previous study demonstrated that endothelial dysfunction serves a vital role in vascular disease which is caused by diabetes mellitus [4]. The endothelium is in direct contact with the bloodstream, meaning it may become a prospective target for drug delivery.

Phosphoinositide 3-kinase (PI3K) may induce endothelial cell migration and proliferation in endothelial cells through manifold signaling pathways. PI3K-protein kinase B (Akt) signaling pathways may be a potential therapeutic method. When the oxidative stress links postprandial hyperglycaemia with endothelial dysfunction, patients with diabetes may present raised oxidant and reduced antioxidant levels [5]. The PI3K-Akt signaling pathway serves a notable role in cell apoptosis and proliferation in addition to other activities. One study confirmed that it possesses a specific role in the pathophysiology of angiopathy [6]. The activated PI3K-Akt signaling pathway has also been demonstrated to increase insulin sensitivity, serve the role of regulating sugar lipid metabolism, and protect the vascular endothelium [7].

Endothelial cells are a key target for diabetic vascular disease. Numerous studies have demonstrated that obesity, insulin resistance, and type 2 diabetes are associated with abnormal endothelial function and participate in the occurrence and development of diabetes mellitus and small vascular complications [8]. Vascular endothelial growth factor (VEGF) is a crucial cytokine used to promote the formation of new blood vessels, which may promote vascular endothelial cells, smooth muscle cell mitosis, vascular permeability, and angiogenesis [9, 10]. Previous studies on the association between VEGF and vascular disease revealed that serum VEGF was significantly increased in patients with diabetes mellitus, and the increase of VEGF was closely associated with the germination of diabetic angiopathy [11, 12]. The present study intended to investigate the vascular lesion of type 2 diabetes mellitus in Goto-Kakizaki (GK) rats and human umbilical vein endothelial cells, in order to illustrate the mechanism of the pathogenesis of the vascular lesion of type 2 diabetes mellitus. It was hypothesized that this may provide a profound understanding of type 2 diabetes mellitus.

## 2. Materials and Methods

### 2.1. Ethics Statement

Male Wistar rats (200 ± 20 g, 5 months old, specific-pathogen-free (SPF) grade) were obtained from the Experimental Animal Center of Anhui Medical University (No. SCXK (Wan) 2011-002; Hefei, China). Spontaneous type 2 diabetes mellitus GK rats (200 ± 20 g, 5 months old, SPF grade) were obtained from the Shanghai Slix Experimental Animal Co. Ltd (No. SCXK (HU) 2012-0002). The study was approved by the ethics committee of the Experimental Animal Ethics Committee of Anhui Medical University, Hefei, China. All surgery was performed under sodium pentobarbital and minimized suffering.

### 2.2. Chemicals

L-Nitro-arginine-methyl-ester (L-NAME) was purchased from Sigma-Aldrich (Merck KGaA, Darmstadt, Germany; batch no. N5751-10), and sodium pentobarbital was purchased from Shanghai Chemical Reagent Co., Ltd. (Shanghai, China). Anti-HIF-1 alpha antibody, anti-PTEN antibody, anti-VEGFA antibody, anti-PI3K p85 antibody, anti-PI3K p85 (phospho Y607) antibody, and anti-AKT1 (phospho S473) antibody were purchased from Abcam (batch no. Ab1; ab32199; ab1316; ab86714; ab182651; ab81283; dilution 1 : 200; 1 : 100; 1 : 100; 1 : 100; 1 : 200; 1 : 200); TRIB3 polyclonal antibody, AKT (L321) polyclonal antibody, and AKT (phospho-T308) polyclonal antibody were purchased from Bioworld (batch no. BS60451; BS1502; BS4647; dilution 1 : 100; 1 : 100; 1 : 100).

### 2.3. Animal Experiments and Sample Collection

All rats were allowed *ad libitum* access to food and water and a separate room in a facility at a temperature of 18-22°C and humidity of 40-60%. The Wistar rats were administered regular feed, and the GK rats were fed a high-fat feed (as follows: normal feed, 88.2%; refined lard, 10%; cholesterol, 1.5%; and pig bile salt, 0.3%). Subsequent to acclimatization for 1 week, the caudal edge of the liver in GK rats exhibited a high blood flow when the rats were awake and the blood glucose of the GK rats was determined using a Roche glucose meter (Roche Diagnostics, Basel, Switzerland). The standard of type 2 diabetes mellitus is blood sugar > 11.1 mmol/l, and the results met these criteria. The rats were randomly divided into a GK control group (*n* = 10), a GK experimental model group (*n* = 10), and a Wistar control group (*n* = 10). The drinking water from the GK experimental model group had L-NAME (0.1 mg/kg) added to it for continuous molding for 42 days. All rats were anesthetized with sodium pentobarbital (30 mg/kg, intraperitoneal injection), and abdominal aortic tissues were sealed and stored at -80°C. All rats were euthanized prior to this isolation of aortic tissue by cervical dislocation.

### 2.4. Cell Culture

Human umbilical vein endothelial cells were cultured in RPMI-1640 medium (Thermo Fisher Scientific, Inc., Waltham, MA, USA) with 10% fetal bovine serum and 1% penicillin/streptomycin (Sigma-Aldrich; Merck KGaA). Cells were incubated under 5% CO_2_ at 37°C; normal medium was 5.5% glucose.

### 2.5. MTT Cell Experiment

The cell seed plate was treated with drug addiction according to the experimental scheme, and the concentration of each sample was set to 3-5 replications. 20 milliliters of l MTT solution (5 mg/ml, i.e., 0.5% MTT) was added to all holes and incubated in the incubator for 4 hours. Carefully remove the supernatant and add DMSO (dimethylsulphoxide) at 150 milligrams per well for 10 min at low speed (120~140 rpm/min) in the shaking table for full dissolution of the crystals. The absorptive value of 490 nm was measured using an enzyme labelling machine, and the inhibition rate of the drug on cells was calculated according to the formula.

### 2.6. Blood Pressure and Heart Rate Analysis

Systolic blood pressure, diastolic blood pressure, mean blood pressure, and heart rate of the rats were monitored using a tail blood pressure measuring instrument, and the mean blood pressure of each animal was measured three times.

### 2.7. Histological Analysis

A right lobe of each aorta abdominalis tissue from each rat was fixed in 10% neutral formalin and stained with hematoxylin and eosin (H&E), Masson, and Verhoeff staining for histological examination.

### 2.8. Immunofluorescence Analysis

Initially, subsequent to drying, the cells were evenly distributed in the center of the cover glass with a group pen; 50-100 *μ*l of the membrane working fluid was added to each dish, incubated at room temperature for 20 min, and then washed with phosphate buffered saline (PBS) for 5 min each time. The tissue was then evenly covered with 3% bovine serum albumin in the ring, and the temperature was maintained for 30 min at room temperature. Following this, the cells were gently shaken with the sealing fluid in the orifice plate, mixed with PBS, and then the cell culture plate was placed flat on a wet box at 4°C for incubation overnight. The cell orifice plate was then placed on the decolorized rocking bed 3 times for 5 min each time. Following drying, the two anticoated tissues of the corresponding species in the inner circle were incubated for 50 min. The slide was then placed in PBS (pH 7.4) and washed three times on the decolorized bed for 5 min each time. Once the section was dried, DAPI dye was added to the ring and incubated for 10 min. The slide was then placed in PBS (pH 7.4) and washed three times on the decolorized bed for 5 min each time. The glass slide was dried and then sealed with antifluorescence quenching. The images were observed and collected under a fluorescence microscope.

### 2.9. Reverse Transcriptase-Quantitative Polymerase Chain Reaction (RT-qPCR) Analysis

TRIzol reagent (Invitrogen; Thermo Fisher Scientific, Inc.) was used to isolate and purify total RNA according to the manufacturer's protocol. The quantity and quality of the RNA samples were determined using the NanoDrop 2000 instrument (Thermo Fisher Scientific, Inc.). The RNA integrity was assessed by electrophoresis with denaturing agarose gel.

RT-qPCR was performed using a fluorescence quantitative PCR instrument (ABI 7300) according to the manufacturer's protocol. *β*-Actin was used as a control. All PCR primers used are listed in Table 1. All experiments were performed in triplicate. The data was analyzed using the 2^-ΔΔCq^ method.

### 2.10. Western Blot Analysis

Cells were scraped and lysed in RIPA buffer (cat no. P0013B; Beyotime Institute of Biotechnology, Haimen, China), and proteins were measured with a bicinchoninic acid protein assay kit (cat no. P0010; Beyotime Institute of Biotechnology). The primary antibodies used in the experiments are as follows: Akt (cat no. BS2987, 1 : 500; Bioworld Technology, Inc., St. Louis Park, MN, USA), VEGF (cat no. ab1316, 1 : 150; Abcam, Cambridge, UK), phosphatase and tensin homolog (PTEN; cat no. ab32199, 1 : 500; Abcam), and *β*-actin (cat no. ab8226, 1 : 2,000; Abcam), followed by horseradish peroxidase-conjugated secondary antibodies (cat no. A0208, 1 : 1,000; Beyotime Institute of Biotechnology). Protein bands were visualized using electrochemiluminescence reagents (cat no. P0018; Beyotime Institute of Technology).

### 2.11. Statistical Analysis

Experimental data were presented as the mean ± standard deviation. Statistical comparisons were performed using Student's *t*-test, and *P* < 0.05 was considered to indicate a statistically significant difference.

## 3. Results

### 3.1. MTT Cell Experiment Result

MTT data showed that high glucose has inhibited cell activity (Table 2).

### 3.2. Blood Pressure and Heart Rate

The heart rate results in the rats revealed that the heart rate of the GK control group was significantly higher compared with in the Wistar control group (*P* < 0.05), but no significant difference was identified when compared with the GK experimental model group. Similarly, systolic blood pressure in the GK control group was significantly higher compared with the Wistar control group (*P* < 0.05), but no significant difference was identified compared with the GK experimental model group.

Additionally, diastolic blood pressure in the GK control group was significantly higher when compared with that of the Wistar control group (*P* < 0.05), but not compared with the GK experimental model group. Finally, the mean blood pressure in the GK control group was significantly higher when compared with that of the Wistar control group (*P* < 0.05), but not when compared with the GK experimental model group (Table 3).

### 3.3. Histopathology

**Figure 1:**
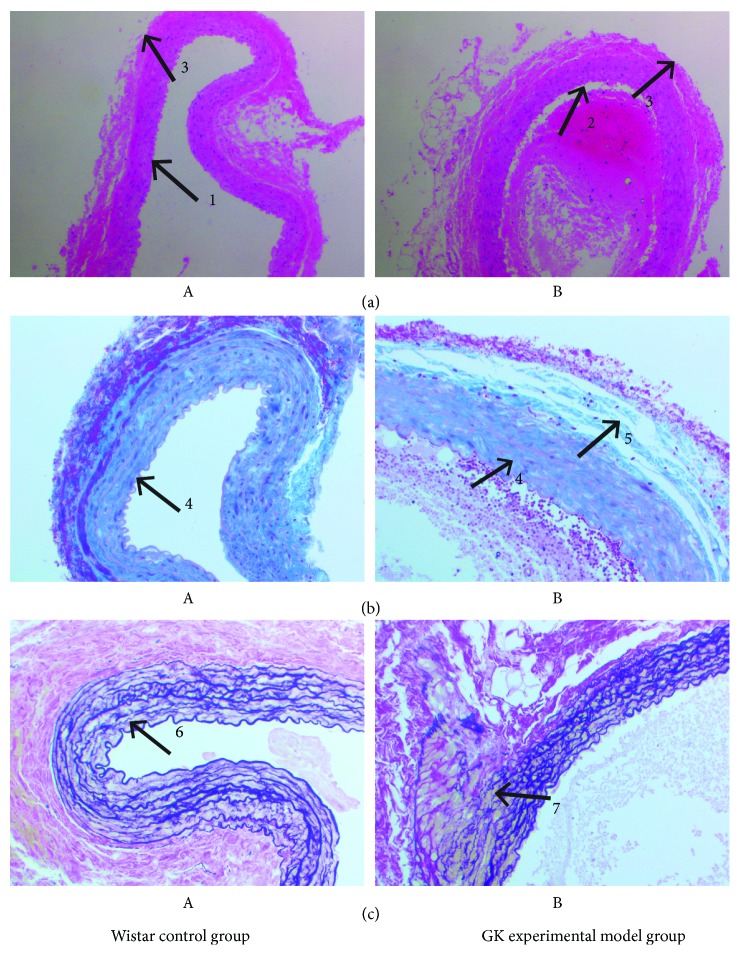
Histological examination of the aorta abdominalis tissue of type 2 diabetes mellitus in rats (magnification ×200). (a) H&E staining; (b) Masson staining; (c) Verhoeff staining; A: Wistar control group; B: GK experimental model group. 1: endothelium cells; 2: swollen and infiltrated endothelium cells; 3: media thickness; 4: collagen fibers; 5: peritubular collagen fibers; 6: elastic fibers; 7: elastic fiber fracture.

H&E staining revealed that the rats in the Wistar control group presented with a flat abdominal aorta intima and flat endothelial cells, clingy on flat in elastic plate, neatly and elastic plate with smooth muscle cells in parallel arrangement, intimal smooth and tidy. Compared with the control group, there was thickening and breakage of the intima, the endothelial cells were partially detached, swollen, and infiltrated in the rats in the GK experimental model group. Additionally, the smooth muscle cells of the medium membrane had undergone hypertrophy, were distorted, and had an arranged disorder, and the number of layers was increased (Figure 1(a)).

Masson staining revealed that the aortic smooth muscle cells and elastic fibers were dyed red and the collagen fibers were blue-green. Rats from the Wistar control group demonstrated that the collagenous fibers of the abdominal aorta wall are evenly distributed and are slender and the adjacent cells have a good network of collagen fibers. The fibrosis of the vessels was very light. Compared with the control group, the collagenous fibers of the abdominal aorta were increased, and the collagen fibers were interlinked into nets or clumps and arranged in a disordered and unevenly distributed manner, tightly surrounding the smooth muscle cells in the rats of the GK experimental model group (Figure 1(b)).

Verhoeff staining revealed that the membrane elastic fibers in the aorta were black or blue-black and the smooth muscle fibers and collagen fibers were red. Rats from the Wistar control group revealed that the abdominal aorta elastic fiber distribution was uniform, neat, and complete without fracture. Compared with the control group, the abdominal aorta elastic fibers were circular, the arrangement was loose, the distribution was unevenly distributed, and the visible fracture was notable in the rats of the GK experimental model group (Figure 1(c)).

### 3.4. Fluorescence Intensity Results

**Figure 2:**
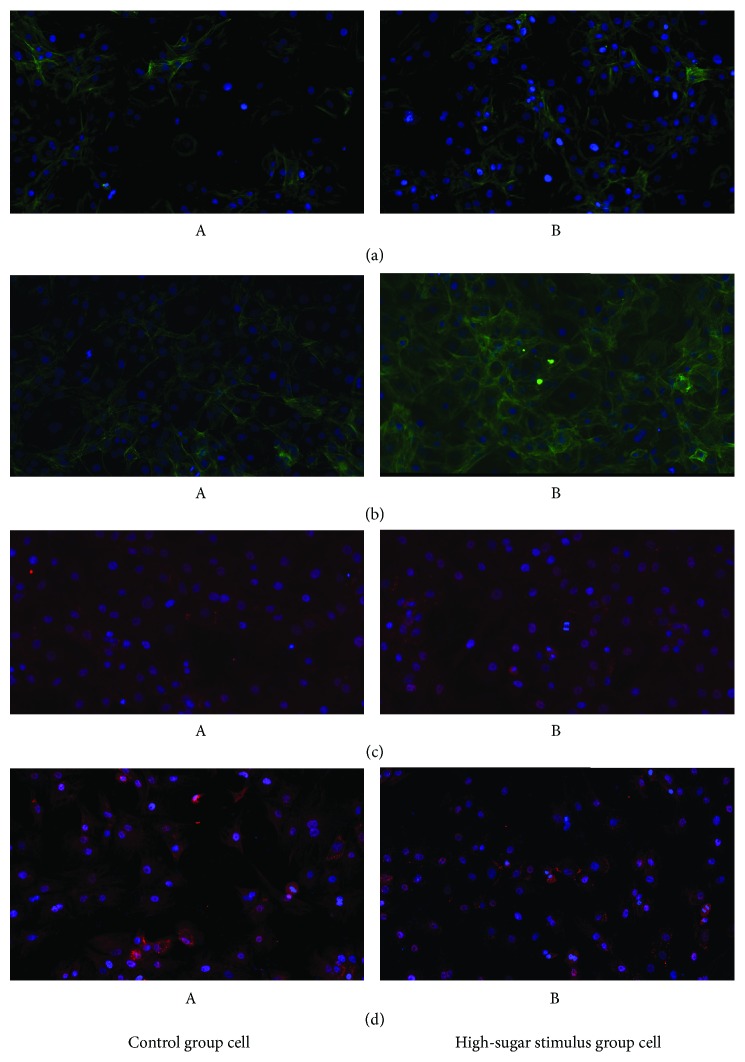
Immunofluorescence analysis of HUVEC cells (magnification ×200). (a) VEGF; (b) AKT; (c) PI3K; (d) PTEN; A: control group cell; B: high-sugar stimulus group.

**Figure 3:**
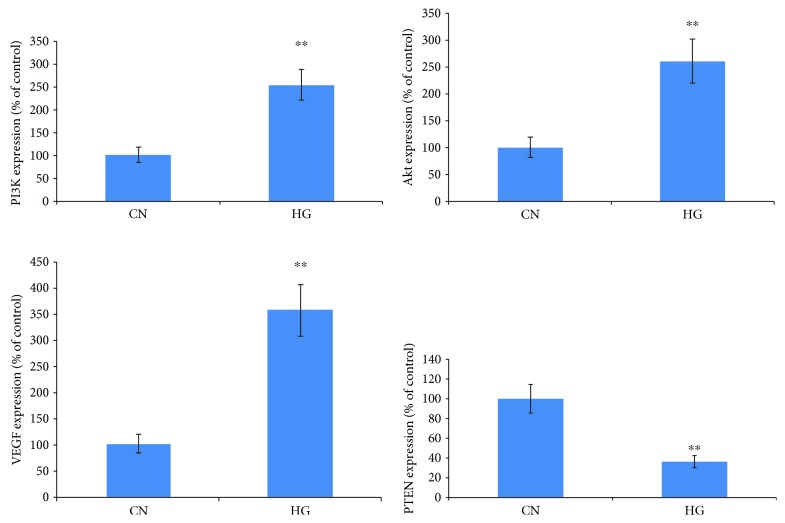
The relative expression of VEGF, AKT, PI3K, and PTEN in immunofluorescence analysis. Compared with the control group, ^∗∗^*P* < 0.01.

Under a confocal microscope, the expression of VEGF, PTEN, PI3K, and Akt in the cells produced blue fluorescence; VEGF in the control group cells produced comparatively weak fluorescence and an enhanced fluorescence intensity in the high-sugar stimulus group. The PI3K and Akt expression in the control group cell demonstrated weak fluorescence and enhanced fluorescence intensity in the high-sugar stimulus group. PTEN in the control group cell demonstrated strong fluorescence, and the fluorescence intensity of the high-sugar stimulus group was comparatively weak (Figures 2 and 3).

### 3.5. MRNA Expression Level Results

The amplification curve revealed that in the present study, the reaction curves of PTEN, Akt, PI3K, and VEGF were substantial, and these exhibited high efficiency rates and good parallelism of amplification curve. Additionally, a repeated reaction produced a similar level of amplification, demonstrating the efficiency, repeatability, and accuracy of this experiment. The low concentration curve index period is obvious, not easy to appear false masculine misjudgment, and had high sensitivity. The melting curve revealed a curve with a single peak, indicating that the amplification product was pure. The product length met the design requirements, indicating that the Cq value produced was accurate.

**Figure 4:**
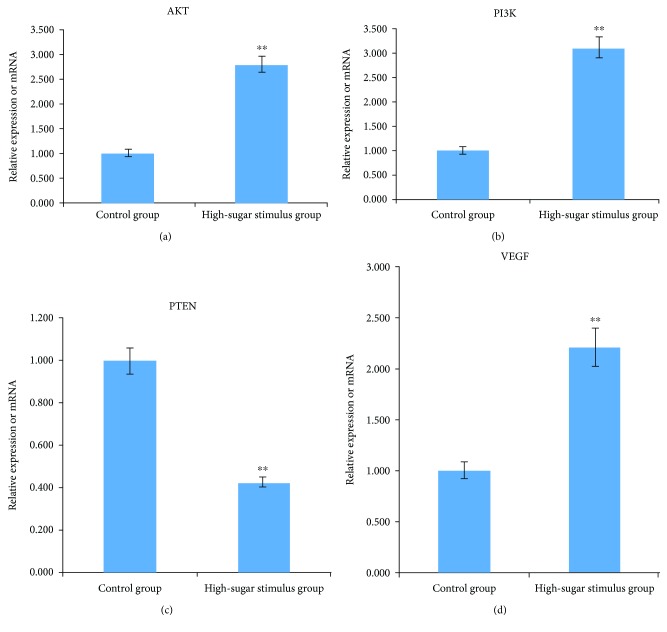
The expression of VEGF, AKT, PI3K, and PTEN mRNA in HUVEC cells. Compared with the control group, ^∗∗^*P* < 0.01.

RT-qPCR revealed that PTEN mRNA expression levels in the high-sugar stimulus group were significantly lower compared with the control group (*P* < 0.01) and that the mRNA expression levels of PI3K, Akt, and VEGF in the high-sugar stimulus group were significantly higher compared with the control group (*P* < 0.01) (Figure 4).

### 3.6. Protein Expression Level Results

**Figure 5:**
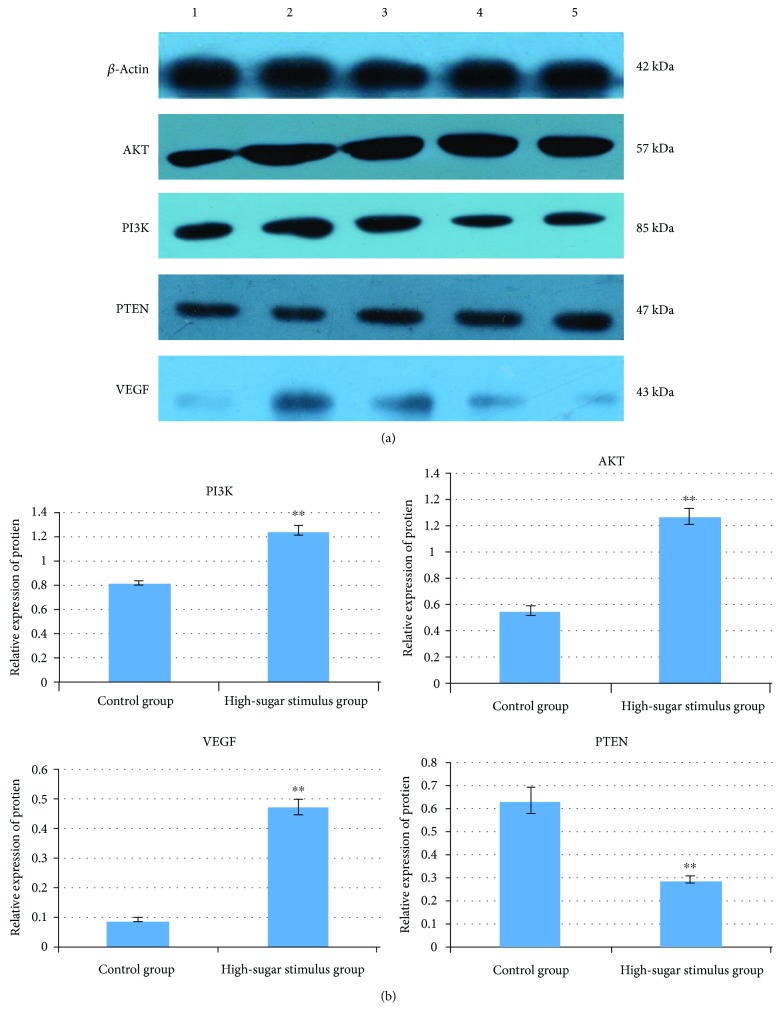
Western blot analysis of VEGF, AKT, PI3K, and PTEN protein (a). The expression of VEGF, AKT, PI3K, and PTEN protein in HUVEC cells (b). Compared with the control group, ^∗∗^*P* < 0.01.

Western blot analysis revealed that the protein expression levels of PTEN in the high-sugar stimulus group were significantly lower compared with the control group (*P* < 0.01) and the protein expression levels of PI3K, Akt, and VEGF in the high-sugar stimulus group were significantly higher compared with the control group (*P* < 0.01) (Figure 5).

## 4. Discussion

GK rats are internationally recognized nonobesity type 2 diabetes mellitus animal models with the following characteristics: lower glucose-stimulated insulin secretion, excessive liver sugar production, and muscle and adipose tissue medium insulin resistance [13, 14]. The administration of the nitric oxide (NO) synthase inhibitor L-NAME for extended periods of time may inhibit endothelial NO synthesis, damage vascular endothelial function, and induce the expression of cytokines including VEGF in order to study animal models of type 2 diabetes mellitus and their large vascular lesions [15, 16]. L-NAME could further induce the vascular disease, which was administered in an animal study. In the present study, the heart rate, systolic blood pressure, diastolic blood pressure, and mean blood pressure were assessed and were revealed to be higher in the GK control group compared with the Wistar control group; and no difference was revealed when compared with the GK experimental model group. H&E, Masson's, and Verhoeff staining revealed that the rats from the GK experimental model group had damage at different levels.

Vascular endothelial cells are highly differentiated monolayer cells which cover the surface of the vascular lumen and serve important functions including regulating vascular permeability, maintaining blood flow, and regulating vascular smooth muscle cell proliferation. Endothelial cell apoptosis may increase smooth muscle cell proliferation and migration, enhance blood coagulation, and increase leukocyte infiltration into the endothelium thus leading to endothelial dysfunction [17, 18]. Multiple metabolic disorders in type 2 diabetes mellitus accelerate this pathophysiological process. In the state of diabetes, numerous factors including high blood sugar levels, glycation end products being produced, ischemia, and hypoxia promote the expression of cytokines associated with angiogenesis [19]. The dysfunction of endothelial cells has become an important initial factor for the development of type 2 diabetes mellitus and its vascular complications. Additionally, atherosclerosis is a type of chronic pathological change in the middle arteries, which is the basis of the formation of a diabetic vascular system [20, 21].

VEGF is a glycosylated mitogen that specifically affects endothelial cells and has various effects, including mediating increased vascular permeability, inducing angiogenesis, vasculogenesis, and endothelial cell growth, promoting cell migration, and inhibiting apoptosis [22–24]. VEGF additionally mediates vascular endothelial permeability and proliferation [25]. In the present study, the fluorescence intensity, protein expression, and mRNA expression of VEGF in the high-sugar stimulus group were higher compared with the control group. These results illustrate that angiogenesis is an important aspect of the pathogenesis of type 2 diabetes mellitus vascular disease.

PTEN is a tumor suppressor which is mutated in a number of different cancer types at a high frequency. Phosphatidylinositol-3,4,5-trisphosphate 3-phosphatase is encoded by this gene. This protein dephosphorylates phosphoinositide substrates unlike other protein tyrosine phosphatases [26]. It functions as a tumor suppressor and regulates intracellular levels of phosphatidylinositol-3,4,5-trisphosphate in cells by negatively regulating the Akt signaling pathway. This also regulates energy metabolism in the mitochondria [27, 28].

Angiogenesis is a tyrosine kinase and its ligand signals through a complicated endothelial molecular system, and this ligand signaling system regulates the endothelial cell function, including endothelial cell proliferation, migration, and capillary formation [29]. The VEGF/VEGF receptor signaling system controls these processes and is closely associated with the PI3K signaling pathway [30, 31]. PI3K is activated on the membrane subsequent to the generation of phosphatidylinositol triphosphate (PIP3), resulting in the activation of downstream proteins including Akt which serve a vital role in the process of angiogenesis. PIP3 is the target molecule of phosphatase action, which is the key component of the cell growth regulation pathway. The main function is to stimulate cell growth and block apoptosis [32, 33]. PTEN produces PIP3 for phosphorylation and inhibits the phosphorylation of the PI3K kinase, thereby blocking the Akt signaling pathway, blocking the downstream enzyme activity, and regulating the endothelial cells' VEGF signaling pathways and cellular response. Therefore, PTEN serves a negative regulatory role in the development of novel blood vessels [34]. By inhibiting the PI3K/Akt signaling pathway, PTEN serves an inhibitory role in the formation and development of the pathological neovascularization of atherosclerotic plaques [35–37].

In the present study, the fluorescence intensity of Akt and PI3K in the high-sugar stimulus group was stronger compared with the control group, and that of PTEN in the high-sugar stimulus group was weaker compared with the control group. Akt and PI3K mRNA and protein expression in the high-sugar stimulus group were higher compared with the control group, and that of PTEN mRNA in the high-sugar stimulus group was lower compared with the control group. These results illustrate that PTEN serves a negative regulatory role in the development of novel blood vessels and may inhibit the PI3K/Akt signaling pathway. It inhibits the formation and development of the pathological neovascularization of an atheromatous plaque.

In summary, when vascular disease occurred in type 2 diabetes rats, the expression of VEGF in the aortic tissue was increased, the expression of PTEN was decreased, and the activity of the PI3K/Akt signaling pathway was decreased. Excessive proliferation of vascular endothelial cells is a common model in diabetic vascular disease. Therefore, inhibiting excessive proliferation of vascular endothelial cells is conducive to the effective control of diabetic vascular disease. This study furthered the understanding of the mechanisms of type 2 diabetes and produced a potential method for future disease treatment studies, which may contribute to the development of novel diagnostic markers and therapeutic targets for the clinic treatment of type 2 diabetes. However, there were still some limitations in the current study. Glucose metabolism is mainly detected when insulin resistance mechanism is performed, while we focus on vascular lesions, so nevertheless, further experiments are required to validate their effects in type 2 diabetes. At the present stage, we mainly want to explain the mechanism of damage to large vessels, especially endothelial cells in the process of diabetes. We are also designing and carrying out the mechanism of the regulation of angiogenesis by antihypertensive drugs.

## Figures and Tables

**Table 1:** Real-time quantitative PCR primer sequence.

Gene name	GeneBank ID	Sense primer	Antisense primer	Product length
Akt	NM_005163	GCACAAACGAGGGGAGTACAT	AGCGGATGATGAAGGTGTTGG	192
PI3K	NM_181523	ACCACTACCGGAATGAATCTCT	GGGATGTGCGGGTATATTCTTC	207
PENT	NM_000314	AGGGACGAACTGGTGTAATGA	CTGGTCCTTACTTCCCCATAGAA	100
VEGF	NM_001171627	AGGGCAGAATCATCACGAAGT	AGGGTCTCGATTGGATGGCA	75
ACTA	NM_001101	CATGTACGTTGCTATCCAGGC	CTCCTTAATGTCACGCACGAT	250

**Table 2:** MTT cell experiment data.

Group	OD average (24 h)	OD average (48 h)	Inhibition ratio (24 h)	Inhibition ratio (48 h)
Normal HUVEC	0.87	1.17	—	—
30 mM high glucose group	0.84	0.94	0.03	0.19
5.5 mM glucose + 24.5 mM mannitol high osmotic pressure group	0.84	0.94	0.03	0.20
5% drug serum	0.85	1.06	0.03	0.09
10% drug serum	0.81	0.92	0.07	0.21
15% drug serum	0.78	0.81	0.11	0.31
20% drug serum	0.68	0.73	0.22	0.38
25% drug serum	0.62	0.54	0.29	0.54
30% drug serum	0.53	0.50	0.39	0.57

**Table 3:** The SBP, DBP, MBP, and HR of rats (, *n* = 10).

	Wistar control group	GK control group	GK experimental model group
SBP	128.37 ± 4.92	185.20±10.06^∗∗^	187.31 ± 10.29
DBP	117.86 ± 5.45	167.45±6.73^∗∗^	162.72 ± 7.93
MBP	124.86 ± 4.14	179.28±6.12^∗∗^	179.11 ± 6.71
HR	327.10 ± 12.16	442.20±17.77^∗∗^	439.00 ± 24.32

Note: compared with the control group, ^∗∗^*P* < 0.05.

## Data Availability

The data used to support the findings of this study are available from the corresponding author upon request.
